# Association between the use of mobile touchscreen devices and the quality of parent-child interaction in preschoolers

**DOI:** 10.3389/frcha.2024.1330243

**Published:** 2024-03-27

**Authors:** Krisztina Liszkai-Peres, Zsófia Budai, Adrienn Kocsis, Zsolt Jurányi, Ákos Pogány, György Kampis, Ádám Miklósi, Veronika Konok

**Affiliations:** ^1^ Department of Ethology, Institute of Biology, ELTE Eötvös Loránd University, Budapest, Hungary; ^2^ Doctoral School of Biology, Institute of Biology, ELTE Eötvös Loránd University, Budapest, Hungary

**Keywords:** preschooler media use, mobile touchscreen devices, parent–child interaction, social interactions, smartphone/tablet use, online/offline activities

## Abstract

The early use of mobile touchscreen devices (MTSDs), including smartphones and tablets, may reduce the frequency and quality of social interactions between children and parents, which could impact their relationship and have negative consequences on children's socio-cognitive development. In this study, we applied a parental questionnaire and a behavioral observational method in a laboratory setting (free and structured play sessions) to examine the association between preschool MTSD use and the quantity and quality of parent–child relationships. Our findings revealed that preschoolers who regularly use MTSDs (*n* = 47, aged 4–7 years, engaging in MTSD use for at least 2 h per week) are spending less time with their parents and exhibited lower quality interactions compared to non-users (*n* = 25). However, shared offline leisure time with parents serves as a protective factor among MTSD-users. Furthermore, our study demonstrated a positive association between parents' and children's media use. The results suggest that preschool MTSD use may have unfavorable effects on parent–child interactions, both in terms of quantity and quality. Alternatively, lower quantity and quality of parent–child interaction may lead to higher MTSD use in the child. Based on the results, the importance of engaging in sufficient offline family interactions besides digital media use should be emphasized to parents of preschoolers, and health organizations and governments should include this in their recommendations and policies concerning childhood digital media use.

## 1 Introduction

Children usually form their first social impressions based on their interactions with their parents, and these early experiences have long-lasting effects on their social, cognitive and emotional development (1–3). For example, characteristics of early parent–child interactions shape the attachment style of an infant (4), that determines how they form relationships with others throughout their entire lives (5). Joint attention serves as the foundation for parent–child interactions, wherein partners pay attention to each other and redirect their focus to align with the other's attention (6). This facilitates the synchronization of actions, thoughts, and emotions (7–10). However, social situations that would entail joint attention are often disturbed nowadays by the constant presence of attention-demanding, interrupting mobile touchscreen devices (*MTSD*s: tablets and smartphones).

The rapid increase of MTSD use is a global phenomenon. As of 2022, approximately 67% of the world's population (5.32 billion people) owned a mobile phone, spending around 6 h per day on the internet (11). These statistics indicate that MTSD use is a time-consuming activity that detracts from other aspects of life, including face-to-face social interactions (also known as the *social displacement hypothesis*) (12–16). Moreover, MTSDs and other digital devices not only affect the quantity but also the quality of social interactions (16). The term *technoference* describes the phenomenon whereby digital technology frequently interrupts our lives through beeping signals, incoming calls, or vibrations (17, 18). The disturbing effect of MTSDs is not limited to adult's social interactions (17): it is also observed in parent–child interactions, as demonstrated by several observational studies (19–26). For example, Lemish et al. (21) conducted a field study at a playground involving 60 families. The results showed that 79% of the parents used their mobile phones at least once during their stay at the playground. Based on the observations, alternated attention between the mobile and the child (*divided engagement*) and total absorbance by the MTSDs (*disengagement*) were found to pose safety risks, and the emotional well-being of the child was also compromised (21). Other studies also supported that parental MTSD use is associated negatively with parent–child interaction quality [for a review see (27)]. What further nuances the picture is that parents' absorption in their devices might serve as an undesirable model to follow (28, 29). In line with this, parents' heavy media use is associated with higher media use in children both in terms of TV (30) and MTSD use (29, 31, 32).

MTSD use in childhood is a booming phenomenon, more and more children use MTSDs around the world [e.g., in the UK (33), in the USA (34)]. Regarding the consequences of early MTSD use studies showed that excessive MTSD use of the child is adversely linked with parent–child relationship (although the causality is unknown) (35, 36). These findings may help explain why children who spend more time with digital media tend to perform worse on socio-cognitive and socio-emotional tests compared to non-user children (29, 37–42), and are more likely to experience relationship problems with their peers [(29) but see (43)]. In contrast, a week-long participation in an outdoor camp without access to MTSDs was associated with improved social perception skills at the end of the program (44). Finally, excessive engagement with digital devices not only distances the child from parents and peers but often leads to tantrums and serious conflicts within the family, further straining relationships (45–47).

To further complicate the already complex picture, the association between parent–child interaction and the use of digital devices by both partners can also be explained by reverse causality. The use of digital devices within a family may serve as an indicator of the family climate (48). For example, in families with loose bonds and less secure attachment, both parents and children may prefer digital devices or activities opposed to share time with each other (36, 49–52). Possible reasons for this behavior include compensating for the lack of social support (49), or coping with difficult emotions (53, 54). Additionally, if the child's MTSD use is unsupervised (how much and what contents they consume), it could end up in higher levels of and/or more problematic digital device use [for a review see (54)]. Therefore, lower parent–child interaction quality and quantity and excessive digital media use can have a bi-directional relation, leading to a vicious circle. Although digital device use might disrupt family interactions even in well-functioning, warm families, the engagement in sufficient joint offline interactions might protect children against becoming problematic MTSD users.

Nevertheless, so far only the parent's digital media use, but not the child's media use was observed in terms of its disrupting effects on the quantity and quality of parent–child interactions. As more and more children use MTSDs, and for an increasing amount of time (29, 55, 56), there is an urgent need to investigate whether this has negative effects on parent–child relationship, as it can have serious consequences on children's socio-emotional and socio-cognitive development.

Moreover, studies investigating MTSD use in childhood mainly focus on school-aged children or adolescents [e.g., (57–60)], despite the fact that more and more children become user at a younger age (29, 61, 62). Although children mainly use MTSDs actively at home, the presence of MTSDs is tend to become a common phenomenon in preschool classrooms, as well (63). It is worth highlighting that the preschool years are a sensitive period of life when the foundation of various cognitive skills are established [e.g., (64–66)]. For example, numerous studies investigate the link between media and MTSD use and the development of attention and executive functions [for a review see (66)]. There are also some studies using experimental design, that could show not only association but a direct effect, as well. In Lillard and Peterson's (67) study watching fast-paced videos lead to worse performance on tasks measuring executive functions in a group of 4-year-olds. Konok et al. (68) also found that playing with digital games affected attention at the age of 4–6 years. A further research area of this topic is whether ADHD (attention deficit hyperactivity disorder) or ADHD-like symptoms are related to early use of MTSDs. Results show a mixed picture, some studies found an association between MTSD use and ADHD (43, 69, 70), while others are not (71). Regarding social and emotional development, parents play a crucial role during the preschool years and before (72), and disturbances in family interactions, such as those related to MTSD use, may have consequences in later life. For example, Hinkley et al. (73) found that families with higher screen time during their child's early years do not support children's well-being as well as other families. Poulain et al. (74) also found that high screen time of mothers was associated with emotional problems. From a methodological point of view, the preschool period may be the last time in children's lives when non-users are available as a naturally formed control group, as non-users become a minority or a special group at later ages (e.g., children in alternative schools where MTSD use is forbidden).

Furthermore, although there is an increasing number of studies investigating the effects of MTSDs on families, particularly regarding parental MTSD use, these studies primarily rely on parental questionnaires or observational data from field studies [for a review see (75)]. There has been a lack of controlled laboratory studies that directly observe and measure parent–child interaction.

### 1.1 Aims, hypotheses (H) and predictions (P)

In the present study, our aim was to investigate the association between the quality and quantity of the parent–child relationship and preschoolers' MTSD use. To achieve this, we compared the parent–child interaction in preschoolers who intensively use MTSDs (MTSD-users as follows) and preschoolers who do not use MTSDs at all (non-users). We were also interested in examining whether the parent–child relationship is linked to the child's problematic MTSD use and conflicts within the family regarding the child's MTSD use.

Based on our main hypothesis (H1) that MTSD use is negatively associated with parent–child interaction quantity and quality, we expected the following outcomes:

• Parent–child dyads in the MTSD-user group would spend less time engaged in shared free-time activities (P1), including joint offline activities (P2), but more or equal time engaged in joint online activities (P3) compared to dyads in the non-user group.
• Parent–child dyads in the MTSD-user group would exhibit lower-quality interactions in the laboratory setting compared to parent–child dyads in the non-user group (P4).
• We also predicted that among MTSD-users, problems related to MTSD use would be more prevalent in families where parents and children spend less time together engaging in offline leisure activities (P5).

Additionally, we hypothesized (H2) that there would be a positive association between parents' and children's digital media use, suggesting that MTSD use in children is associated with that of their parents.

## 2 Material and methods

Our study is part of a longitudinal experimental study containing two test sessions with a 2-month delay between them, in which the effect of an educational application was compared among three groups (an experimental MTSD-user group, an MTSD-user control group and a non-user control group). In the current study, we analyzed only the first session of the experiment (i.e., before any experimental treatment had occurred) and we merged the two MTSD-user groups in the analyses as we were interested in the differences between MTSD-users and non-users.

In the presented study we utilized a combination of questionnaire methods and controlled behavioral observations in a laboratory setting.

As children were randomly assigned to the experimental MTSD-user group and the control MTSD-user group, the two subgroups were similar in terms of any potential confounding effects before the treatment.

### 2.1 Participants

A total of 72 parent–child dyads participated in the study, with each dyad consisting of a child (39 boys and 33 girls) and one of their parents (10 fathers and 62 mothers). The inclusion criteria for the children encompassed an age range of 4–7 years, typical development without any developmental or psychiatric diagnoses, and specific digital activity parameters, which were assessed during the recruitment phase using a screening questionnaire (Supplementary Material Appendix 1).

In the non-user group, children were included if they had actively used MTSDs (passive use, such as watching videos, was not considered as an exclusion criterion) fewer than 5 times in their lifetime, according to the parents' responses. In the MTSD-user group, children were included if they met the following criteria: (1) using digital devices for a minimum of 2 h per week, (2) having a usage duration of at least 6 months, and (3) actively using the device, such as playing games on it.

The non-user group comprised 25 children (15 boys and 10 girls; mean age ± SD = 5.22 ± 0.69 years; range = 4.2–6.8 years), while the MTSD-user group comprised 47 children (24 boys and 23 girls; mean age ± SD = 5.38 ± 0.77 years; range = 4–6.8 years). The uneven sample sizes were a result of the study being part of a larger experimental study (as mentioned above). For more information about the demographic characteristics of the two samples see Supplementary Material Appendix 4.

Participants were recruited through online advertisements, and they received a small gift (e.g., pencils, toy cars, doll accessories, etc.) as a token of appreciation for their contribution. Data collection took place between September 2019 and August 2021 in Budapest, Hungary.

#### 2.1.1 Ethical statement

Parents gave written informed consent, and before the experiment the experimenter explained the tasks to the children and their right to disrupt the study or take a break any time. The study was carried out according to national and international ethical standards (The Code of Ethics of the World Medical Association; Declaration of Helsinki) and was approved by the United Ethical Review Committee for Research in Psychology (EPKEB, permission no. 2019/17).

### 2.2 Materials and procedure

All tests were conducted in a child-friendly laboratory at the Eötvös Loránd University in Budapest, Hungary. The experiments were administered by one of five professional experimenters. Prior to the start of the test session, the experimenter provided information about the study to both the parent and the child, and written consent was obtained from the parent while oral consent was obtained from the child. The test session started with the Parent–Child Interaction Test. Following the interaction test, parents were asked to complete the Digital Media Use Questionnaire online using a tablet, while their children participated in behavioral socio-cognitive tests that are not part of the current study.

#### 2.2.1 Digital media use questionnaire (DMUQ)

We created the Digital Media Use Questionnaire (DMUQ) for this study partly based on the study of Konok et al. (29), consisting of 31 questions (Supplementary Material Appendix 2). The questionnaire has four main sections: (I) Demographic data of the family and parental digital behavior in the presence of the child; (II) Child's digital media use; (III) Problematic MTSD use; and (IV) Shared free time activities (online and offline). In the following description we highlight the questions involved in the current analysis.

##### 2.2.1.1 Demographic data and parental digital media use in the presence of the child

Parents participating in the study were asked e.g., about their age, gender, highest level of education, and about the family's monthly net income (the answer was optional). Parents were also asked about their average daily use of TV, mobile phone, laptop/PC, and tablet in the presence of their child (in h and min). This section contained 13 questions. Most of the collected data did not require any conversation, only the average daily use was converted to h from the h and min format.

##### 2.2.1.2 Child's digital media use

Questions were asked about the child's average daily use of TV, laptop/PC, mobile phone and tablet (in h and min). The answers were later converted to only hours. This section contained 7 questions.

##### 2.2.1.3 Problems related to MTSD use

This section was displayed only for participants in the MTSD-user group.

Four questions measured, with a 5-point Likert scale, the frequency of conflicts about MTSD use, e.g., tantrums because of shutting down the device. Internal reliability of the four items was good (Cronbach Alpha = 0.8). An “MTSD conflict” scale was then created by summing up the scores of the four items (resulting in a range of 5–20 scores for this variable).

Likewise, four questions measured the child's behavior indicative of problematic MTSD use with a 5-point Likert scale, e.g., “My child wants to use MTSDs all the time”. Internal reliability of the four items was high (Cronbach Alpha = 0.91), and again, responses were summed to create a “problematic MTSD use” scale with a range of 5–20 scores.

##### 2.2.1.4 Shared free time activities between the parent and the child

Parents were asked five questions about the shared offline and online leisure activities with their child (such as types of joint activities, time spent together at weekdays/weekends). Shared online and offline leisure time variables were created from the weighted average of weekday and weekend data (5 × weekday + 2 × weekend, divided by 7).

#### 2.2.2 Parent–child interaction test (PCIT)

Parent–child interaction was investigated in two sessions: in a 5-min long free play session [based on (76)] followed by a 5-min long structured play session [based on (77)]. During the free play session, the experimenter left the room, but during the structured play session, she stayed for offering help if the participants had any problems.

##### 2.2.2.1 Free play

**Figure 1:**
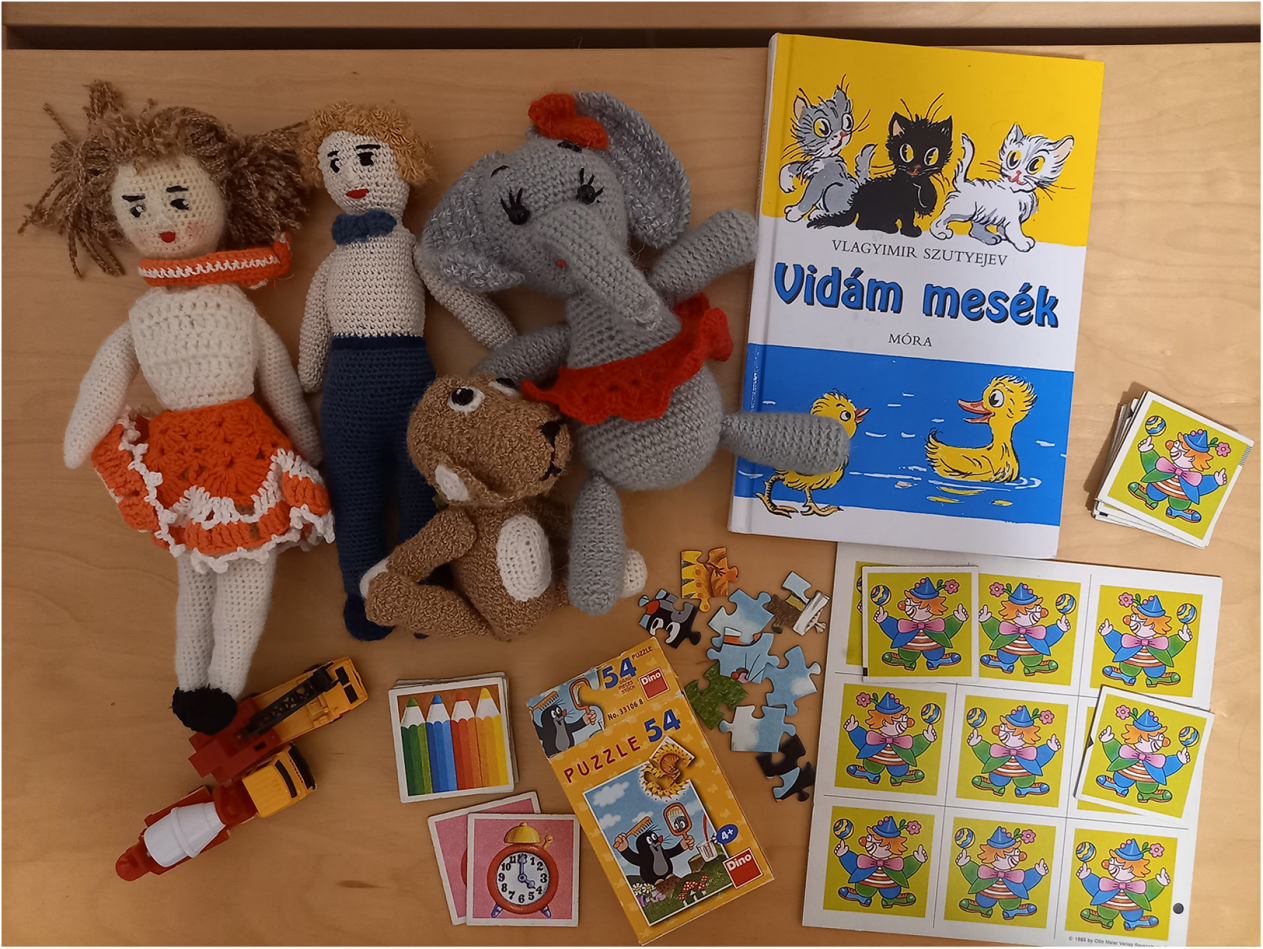
Object set A offered for free play during the first session of the parent–child interaction test.

Before the experiment one of two sets of toys (Set A and B, randomly assigned to the dyads) was put on a table; each set contained a storybook, memory cards, another card game, a puzzle, two toy cars, and 4 puppets (Figure 1). The experimenter showed the toys to the parent–child dyads and asked them to play with them for 5 min (until the experimenter returns). Then the experimenter started the video recording, left the room, and came back 5 min later.

##### 2.2.2.2 Structured play

**Figure 2:**
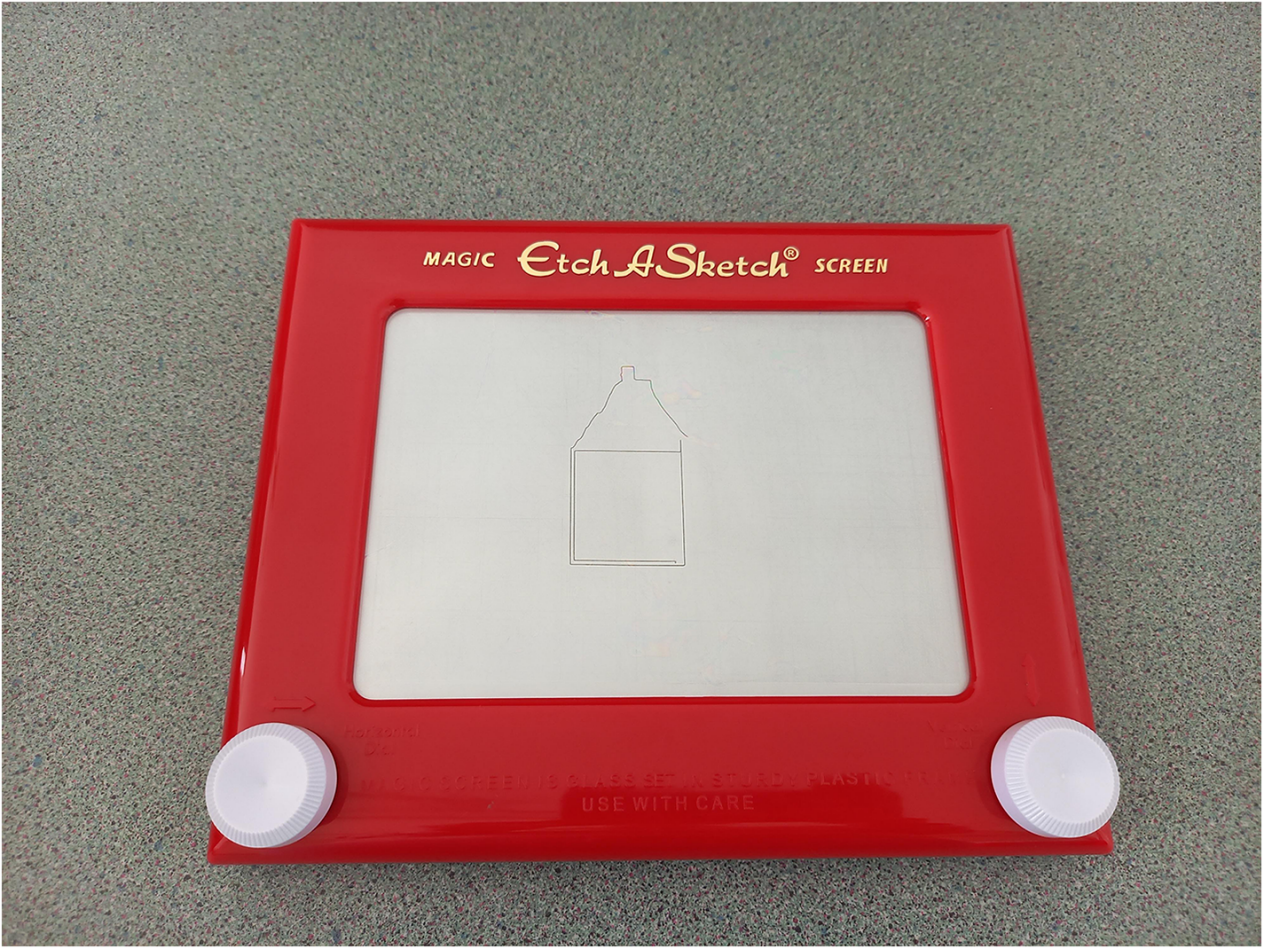
“Etch a sketch” game, used for the structured play during the second session of the parent–child interaction test.

In the structured play task, we used a drawing toy named “Etch a sketch” (Figure 2). This toy consists of a board with two buttons and a screen. The left button controls vertical movement of a line on the screen, while the right button controls horizontal movement. Simultaneously turning both buttons results in a diagonal line. Prior to the task, the dyads were provided with an explanation of how the toy works. They were then instructed to each control one of the buttons (e.g., child controls the left button, parent controls the right button) and collaboratively draw a pine tree (Task A) or a house (Task B). The assignment of tasks was randomly determined for each dyad. Both tasks required the parent and child to synchronize their movements and cooperate in drawing diagonal lines. If the dyads completed their assigned drawing in under 5 min, they were given the option to continue playing and draw anything they desired. The session was video recorded (drawings were not evaluated for the study, only behaviors displayed during the task).

### 2.3 Coding

Video recordings were analyzed using Solomon Coder (© András Péter). Through an exploratory video analysis, we identified recurring actions that could indicate the quality of parent–child interaction. In total, 36 variables were created: 19 behavioral variables for the Free play session and 16 behavioral variables for the Structured play session. (Supplementary Material Appendix 3 provides a comprehensive list of variables and their definitions).

Some of the variables were categorized as *instant*, meaning that we coded only the occurrence of the action, and these occurrences were summed up to obtain a frequency count variable. Other variables were classified as *continuous* where the duration of the action was measured, and a time percentage was calculated. This percentage represents the proportion of the entire session that participants spent engaged in the given action.

To ensure reliability, six coders who were blind to the grouping of the dyads underwent training for video coding. Each coder was responsible for coding a specific number of videos: Coder 1 coded *N* = 2 videos, Coder 2 coded *N* = 7 videos, Coder 3 coded *N* = 18 videos, Coder 4 coded *N* = 14 videos, Coder 5 coded *N* = 19 videos, and Coder 6 coded *N* = 8 videos. Inter-rater reliability was assessed for 20% of the videos, with two coders independently coding the same videos. The results indicated satisfactory reliability. Cronbach's Alpha for the instant variables ranged from 0.72 (Action by child [fp]) to 1 (No answer [fp]), while Cronbach's Alpha for continuous variables ranged from 0.77 (Joint attention [fp]) to 0.91 (Child laughs [fp]).

### 2.4 Statistical analysis

IBM SPSS for Windows, Version 28.0 (Armonk, NY: IBM Corp.) was used for statistical analysis.

Normality tests (Shaphiro–Wilk) were conducted to analyze demographic and media use characteristics of MTSD-users and non-users (see results in the Supplementary Material Appendix 5).

Independent samples *t*-tests were used to compare MTSD-users and non-users regarding parental education, monthly net income, TV watching and shared offline and online leisure time, both separately and summarized (shared offline and shared online time together).

Principal Component Analysis (PCA) with Varimax rotation was used to reduce the number of behavioral variables in the Parent–Child Interaction Test and identify dimensions of parent–child interaction quality. Variables of free play and structured play were involved together in the analysis. A variable was retained if it had 0.4 or higher loading on the respective principal component. Items with a 0.4 or greater loading on more than one component were considered as cross-loadings and were removed. The number of final components was determined based on both the eigenvalues (greater than 1) and the scree plot.

Generalized Linear Models (GzLMs) were applied for investigating the association between each principal components (dependent variables) of the PCIT and experimental group (MTSD-user/non-user) as independent variable. The following potential confounding variables were also included in initial models: parent age, parent gender, parent education, parent net income, child age, child gender, existence of older sibling(s), shared digital activity, shared offline activity, freeplay set (A/B), and structured play task (A/B).

In MTSD-users (*N* = 48), ordinal logistic Generalized Linear Models (ordinal GzLMs) were used to identify the possible associations between problems related to MTSD use (MTSD conflict and problematic MTSD use; dependent variables in separate models) and other variables from the DMUQ (child's gender, child's age, parent's gender, parent's age, parent's education, child's media consumption [summarized], shared offline activities and shared online activities) as independent variables. In all models (GzLMs and ordinal GzLMs), stepwise model selection with backwards elimination was used based on *p*-values.

Associations between parents' and children' media consumption (average daily use) were analyzed separately for all devices (TV, mobile phone, tablet) and together as a total media consumption, using bivariate correlation analysis (Spearman).

## 3 Results

### 3.1 Demographic and media use differences between MTSD-users and non-users (based on DMUQ))

#### 3.1.1 Comparison of indicators of the socioeconomic status (SES) between MTSD-user and non-user groups

We compared whether there is a difference between the two groups regarding the education of the parents and the families’ monthly net income. The groups differed in parental education (*U* = 357.5, *p* < .01). Results showed that parents in the non-user group (M ± SE = 3.77 ± 0.97) were more educated than parents in the MTSD-user group (M ± SE = 3.07 ± 1.12). In the monthly net income there was not any difference between the two groups (*t*_62_ = −1.44, *p* = .078; MTSD-users: M ± SE = 531 714 HUF ± 210 023 HUF; non-users: M ± SE = 617 272 HUF ± 254 804 HUF).

#### 3.1.2 Comparison of TV watching between MTSD-user and non-user groups

Regarding TV watching MTSD-users watch TV more than non-users (*t*_70_ = −3.34, *p* < .01).

### 3.2 Child's MTSD use and the quantity of parent–child interactions (based on DMUQ)

**Figure 3:**
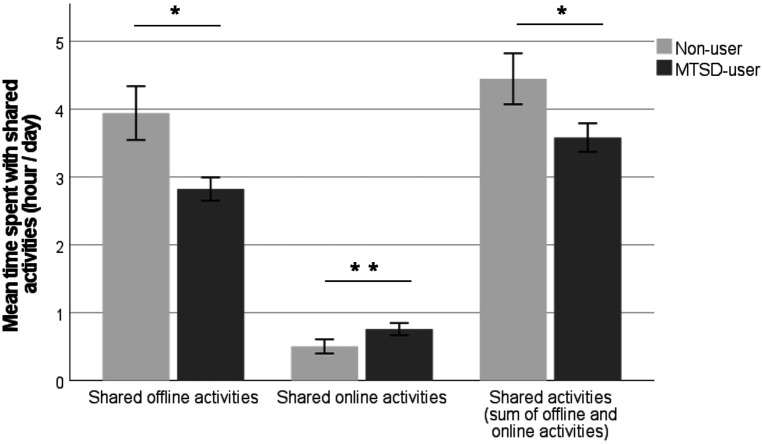
Mean time + SE (h/day) spent together with offline and online activities (separately and in total) in non-users and MTSD-users. Significant results are indicated with asterisks (**p < .01; *p < .05; ns = non-significant, *p* > .05).

Non-user parent–child dyads spent more time (M ± SE = 4.45 ± 0.37 h/day) with joint leisure activities compared to dyads in the MTSD-user group (M ± SE = 3.58 ± 0.21 h/day; *t*_70_ = 2.18, *p* = 0.033; P1). This was due to non-user dyads spending more time with joint offline activities (non-user M ± SE = 3.94 ± 0.39 vs. MTSD-user M ± SE = 2.82 ± 0.17 h/day; *t*_70_ = 2.83, *p* = 0.006; P2) as opposed to spending less time with shared online activities (non-user M ± SE = 0.5 ± 0.1 vs. MTSD-user M ± SE = 0.76 ± 0.08 h/day; *t*_70_ = −2.10, *p* = 0.039; P3) (Figure 3).

### 3.3 Child's MTSD use and the quality of parent–child interaction (based on PCIT)

#### 3.3.1 Principal component analysis of the parent–child interaction test

The PCA of the Parent–Child Interaction Test resulted in 5 components, which explained 61% of the total variance. The five principal components were interpreted as *shared fun* (e.g., both parent and child are laughing), *interactivity* (e.g., the child initiates actions, and the parent responds to it), *parental control* (parent directs the attention of the child verbally and physically), *attention towards partner* (e.g., parent and child look at each other during tasks), and *collaboration* (working together on the task) (see Supplementary Material Appendix 6 for items and their loadings on the respective components).

#### 3.3.2 Association of child's MTSD use and parent–child interaction quality

##### 3.3.2.1 Shared Fun

Only child's age had a marginal positive effect on *shared fun* (B ± SE = 0.01 ± 0.004, *χ*^2^_1, 63_ = 3.71, *p* = 0.054). The other variables (including MTSD use) had no significant effect on the *shared fun* component (all *p* > 0.075).

##### 3.3.2.2 Interactivity

**Figure 4:**
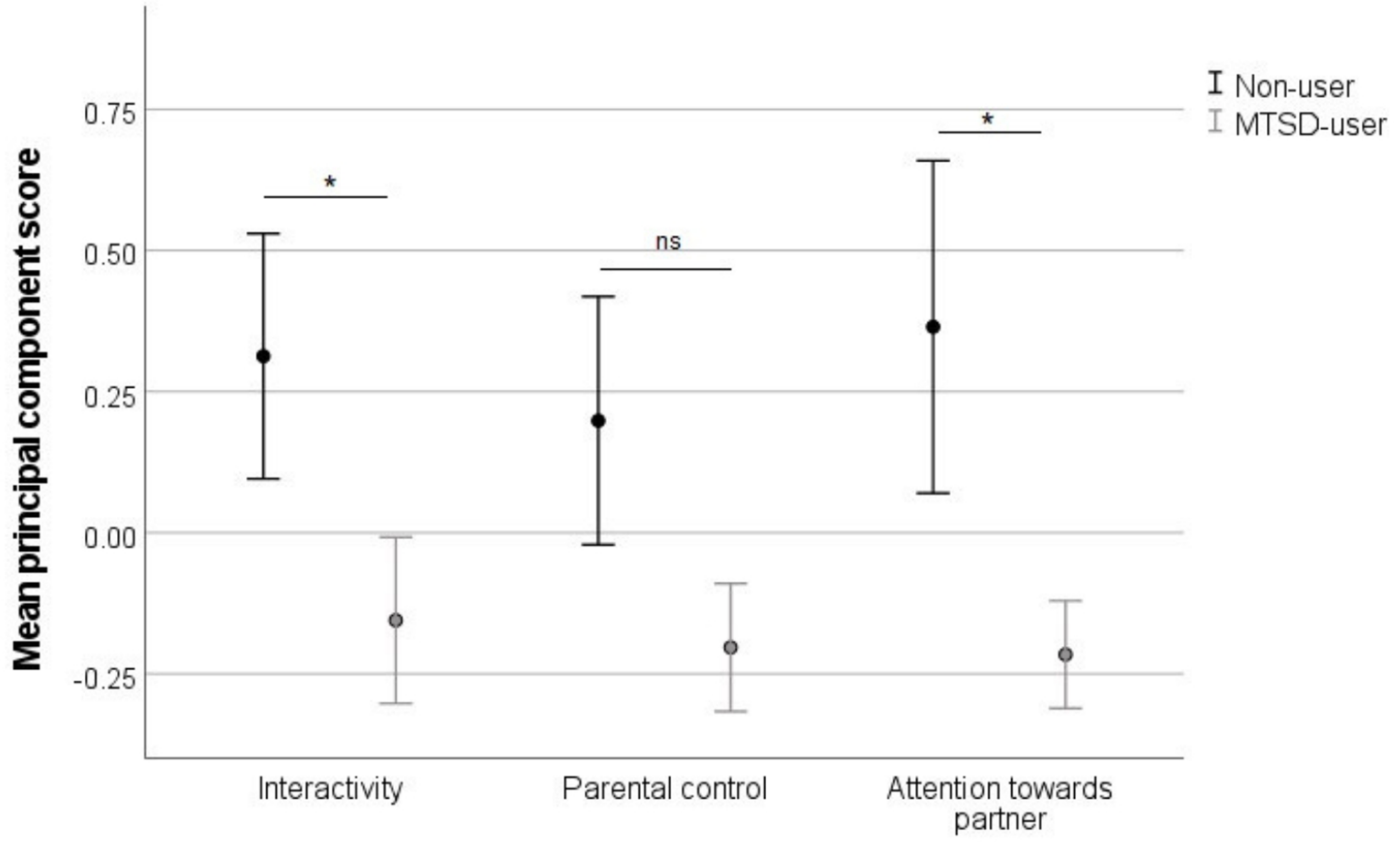
Mean scores and error bars of the interactivity, parental control and the attention towards partner principal components in the non-user and MTSD-user groups. Significant results are indicated with asterisks (**p < .01; *p < .05; ns = non-significant, *p* > .05).

Parent–child dyads in the non-user group were more interactive during shared play sessions than dyads in the MTSD-user group (B ± SE = 0.08 ± 0.03, Wald *χ*^2^_1, 63_ = 7.45, *p* = 0.006). Shared online activity (B ± SE = −0.23 ± 0.09, Wald *χ*^2^_1, 63_ = 6.14, *p* = 0.013), and parent's education were both negatively associated with the interactivity component (B ± SE = −0.43 ± 0.01, Wald *χ*^2^_1, 63_ = 9.37, *p* = 0.002). Parent's gender had a significant effect due to fathers having higher interactivity scores than mothers (B ± SE = 0.12 ± 0.04, Wald *χ*^2^_1, 63_ = 9.34, *p* = 0.002). Task B in structured play was associated with higher scores on interactivity (B ± SE = −0.69 ± SE 0.03, Wald *χ*^2^_1, 63_ = 6.16, *p* = 0.013). (This fact did not influence the results as the ratio of children receiving A and B task was the same in users [24:23] and in non-users [12:13].) The other variables had no significant effect on the Interactivity component (all *p* > 0.24) (Figure 4).

##### 3.3.2.3 Parental control

Parents in the non-user group tended to show more control than parents in the MTSD-user group, although this effect was not significant (B ± SE = 0.03 ± 0.02, Wald *χ*^2^_1, 63_ = 2.9, *p* = 0.088). Structured play task A was associated with more parental control (B ± SE = 0.04 ± 0.02, Wald *χ*^2^_1, 63_ = 4.42, *p* = 0.035) than task B. The other variables had no significant effect on the Parental control component (all *p* > 0.28) (Figure 4).

##### 3.3.2.4 Attention towards partner

Dyads paid more attention towards each other in the non-user than in the MTSD-user group (B ± SE = 0.04 ± 0.02, Wald *χ*^2^_1, 63_ = 3.92, *p* = 0.048). Shared online activity had a positive (B ± SE = 0.13 ± 0.07, Wald *χ*^2^_1, 63_ = 3.84, *p* = 0.05), whereas child's age had a negative effect on this component (B ± SE = −0.29 ± 0.01, Wald *χ*^2^_1, 63_ = 4.79, *p* = 0.029). The other variables had no significant effect on the Attention towards partner component (all *p* > 0.123) (Figure 4).

##### 3.3.2.5 Collaboration

None of the investigated variables explained variation in the Collaboration component (all *p* > 0.113).

### 3.4 Problems related to MTSD use and the quantity of parent–child interactions (based on DMUQ)

#### 3.4.1 MTSD conflict

Analyzed in MTSD-users (*N* = 48), shared offline activities were negatively associated with MTSD conflict score (B ± SE = −1.77, ±0.68, Wald *χ*^2^_1, 48_ = 6.74, *p* = 0.009). Parents' education had a positive effect on MTSD conflict scale (B ± SE = 0.6 ± 0.24, Wald *χ*^2^_1, 48_ = 6.54, *p* = 0.011), so that higher educated parents experienced more conflicts about MTSD use. The other variables (child's gender, child's age, parents' gender, parent's age, child's media consumption, shared online activities) had no significant effect on MTSD conflict score (all *p* > 0.296).

#### 3.4.2 Problematic MTSD use

Shared offline activities were negatively associated with the problematic MTSD use score (B ± SE = −1.64 ± 0.69, Wald *χ*^2^_1, 48_ = 5.66, *p* = 0.017). Both parent's education (B ± SE = 0.93 ± 0.29, Wald *χ*^2^_1, 48_ = 10.44, *p* < 0.001), and child's total media consumption (Wald *χ*^2^_1, 48_ = 5.16, *p* = 0.023) had significant positive effect on problematic MTSD use. The other variables (child's gender, child’ age, parents' gender, parent's age, shared online activities) had no significant effect on problematic MTSD use score (all *p* > 0.098).

### 3.5 Associations between digital media use of the parent and the child (based on DMUQ)

The child's total media consumption was significantly correlated with that of the parent (Spearman's *r* = 0.63, *p* < 0.001). When analyzing the correlations separately for each device, only time spent on watching TV correlated between the parent and the child (Spearman's *r* = 0.59, *p* < 0.001). The use of other devices was not correlated (all *p* > 0.13).

## 4 Discussion

In our study, associations between preschool MTSD use and the quantity and quality of parent–child relationships were revealed via questionnaire method and also in an observational, laboratory environment. Although the applied method is not suitable for describing the possible direct effects of childhood MTSD use on relationships, the results emphasize the importance of investigating childhood MTSD use as a factor regarding the quantitative and qualitative evaluation of family relations. Moreover, the timing of this investigation should start as early as possible, as based on the results the influence of MTSD use is present already during the preschool years, if not earlier [e.g., see (78–80)].

Generally, our results also highlighted the role of shared offline activities as these activities could be considered as a protective factor against problematic MTSD use, and a facilitator of forming high quality relationships among family members. It is worth to note that based on our results shared online activities seem to be less effective despite of the shared component.

### 4.1 Child's MTSD use and the quantity of parent–child interactions

Parent–child dyads in the MTSD-user group spent less time engaging in joint leisure activities, including fewer offline activities, but they spent more time participating in shared digital activities compared to dyads in the non-user group. This finding supports the social displacement hypothesis, suggesting that children who use MTSDs may have less time available for other activities, including offline activities with family members. Additionally, this study corroborates previous research indicating that children who use MTSDs tend to have parents who also engage in higher levels of digital media use [see also (32, 81, 82)]. Consequently, both the child and the parent may have less time dedicated to offline social activities. Furthermore, reduced social interactions within the family, which can indicate lower quality relationships, might lead to increased digital media use as a compensatory or coping strategy (31).

Although dyads in the MTSD-user group spent more time engaging in shared online activities compared to dyads in the non-user group, the overall average time spent on shared online activities for both groups was considerably lower than the time spent on shared offline activities. Therefore, the additional time spent on joint online activities by users compared to non-users is relatively insignificant (the mean for users is only 0.26 h higher than that of non-users), while non-users spend over 1 h more per day on joint offline activities compared to MTSD users. Additionally, research suggests that online/digital parent–child activities are generally of lower quality than offline activities (83) indicating that children in the MTSD-user group are likely participating in fewer high-quality social interactions that are essential for the development of secure attachment and socio-cognitive skills (84, 85).

### 4.2 Child's MTSD use and the quality of parent–child interaction

Our laboratory test results indicate that parent–child dyads in the MTSD-user group exhibited lower-quality interactions compared to dyads in the non-user group. Specifically, we observed differences in three out of the five dimensions of parent–child interaction related to quality: interactivity, attention towards the partner, and marginally parental control.

#### 4.2.1 Interactivity

Children in the non-user group demonstrated higher levels of initiation in interactions, such as asking questions, seeking the parent's attention, and providing instructions, while parents in this group responded more frequently and warmly, including praising the child. These findings align with previous research that suggests a link between digital media use and lower quality interactions (16, 46). However, it remains unclear whether MTSD use is a cause, an effect, or simply a symptom of less strong family relationships. Additionally, it is plausible that engaging in MTSD use as a solitary and time-consuming activity adversely impacts the development of socio-emotional and socio-cognitive skills by displacing social interactions and non-digital play, thereby further hindering the establishment of relationships with others (68, 86).

Furthermore, the results indicated a negative association between shared online activity and the *interactivity* component of parent–child interaction. This suggests that online activities, even when shared with the parent, cannot compensate for the quality of offline shared time. Online and offline shared activities differ significantly: when media is involved, parents tend to be more passive (16, 87). Shared video watching invokes less interaction compared to reading a book or a role play game (88). Parents who co-use media with children, give fewer verbal utterances during electronic play compared to toy play or reading (78, 87, 89–91). This could be also true for digital games, where the parent might only watch how the child plays, but not participate in it actively (92). During joint offline activities, parental scaffolding (assistance) may create opportunities for high quality interactions, such as asking questions, labelling objects and being verbally affectionate (93). However, digital devices themselves may offer children suggestions and feedback to scaffold children's use (94), making parental scaffolding less needed (83). Additionally, younger generations are often more proficient in using digital technology (“digital natives”) than their parents' generation (“digital immigrants”) [for a review see (95)], which further restricts parental scaffolding opportunities.

Surprisingly, parent's education was negatively associated with the *interactivity* parameter. Higher-educated parents may exhibit higher levels of perfectionism and sensitivity to social acceptance (96, 97), which could have made the testing environment more uncomfortable and stressful for them, resulting in lower quality interactions.

Lastly, parent's gender was also associated with the *interactivity* component, with father-child dyads demonstrating higher levels of interactivity compared to mother–child dyads. This finding aligns with previous research of Lindsey et al. (98), which showed that fathers tend to be more initiative and provide more polite commands and imperatives during play with their children compared to mothers. However, it is important to note that the limited representation of fathers in our study (only 10 fathers) raises the possibility of non-representative sampling regarding fathers.

#### 4.2.2 Attention towards partner

Consistent with the findings on *interactivity*, the results also support the social displacement theory, as *attention towards partner* was higher in the non-user group compared to the MTSD-user group. The reduced parent–child interactions due to MTSD use may have a negative impact on the attachment relationship between the child and the parent, as reflected in the diminished dyadic attention, which is a fundamental aspect of social relationships (99). Dyadic attention is crucial for sharing emotions and achieving intersubjectivity, forming the basis for socio-cognitive and socio-emotional development (100). Alternatively, weaker connections (as expressed also by less dyadic attention) among the family members may lead to increased digital device use (31), also in case of the child.

The item *joint attention* loaded negatively on the *attention towards partner* component, indicating that dyads in the MTSD-user group not only spent less time paying attention to each other but also spent more time engaged in joint attention. This may appear contradictory, as joint attention is typically regarded as an indicator of intersubjectivity and a key aspect of social relationships. However, in our coding, we considered behaviors as *joint attention* when both the child and the parent looked at the toy simultaneously. Therefore, these behaviors and *attention towards partner* are mutually exclusive, explaining the opposite loadings on the same principal component. Furthermore, our definition of *joint attention* did not include gaze alternation between the object and the partner, but solely focused on joint attention to the toy. As a result, this behavior may not necessarily indicate a strong social connection, but rather suggests that dyads in the MTSD-user group may prioritize focusing on the object of play rather than on each other.

Interestingly, shared online activity was positively associated with the *attention towards partner* component, seemingly contradicting the previous result (negative link between the child's MTSD use and attention towards the partner) and the findings regarding the *interactivity* component (i.e., that shared online activity was negatively associated with the *interactivity* component). It is possible that even though online activities may not facilitate direct interaction between partners, the physical closeness inherent in shared online activities may enhance attention towards the partner. Additionally, shared online activities cover several different activities (e.g., co-viewing TV might have very different effect on social interactions than co-playing digital games), which the present study does not separate. In addition, while the *interactivity* component has strong association with both the child's MTSD use and the shared online activities, the *attention toward partner* component's association with these variables were barely significant, thus, it should be interpreted cautiously.

#### 4.2.3 Parental control

*Parental control* was slightly higher in the non-user group compared to the MTSD-user group. This may indicate that parents of non-user children are generally more concerned and restrictive, not only regarding MTSD use but also in other areas of their children's lives. This hypothesis could be supported by the result showing that parents of non-users were more educated than parents of MTSD-users. It is possible that higher educated parents have more information about the adverse effect of MTSD use resulting stricter parental rules for the benefit of the child. Generally, higher parental control regarding digital media consumption, including TV watching, internet use, and MTSD use, have been found to be associated with lower screen time, internet use, and MTSD use among children (29, 101, 102).

According to the theory of Baumrind (103), control is one of the dimensions that determine parenting style, with the other dimension being warmth or responsiveness, which is also indicated by the components of *interactivity* and *attention towards partner* in the non-user group. The combination of high demands and responsiveness characterizes authoritative parenting style. Authoritative parents respect their child's opinions while maintaining clear boundaries. They foster their child's demands through bidirectional communication, such as explaining rules, and encourage independence. Authoritative parenting style is associated with the most favorable developmental outcomes for children (104). Therefore, while the MTSD use of family members may decrease the quality of parent–child interactions, the reverse relationship is also highly plausible. In this scenario, a favorable parenting style leads to higher quality parent–child interactions, including more secure attachment, which, in turn, promotes healthier behavior, such as reduced or delayed MTSD use in early childhood or less problematic digital device use in later stages of development (105, 106).

#### 4.2.4 Shared fun and collaboration

We did not find any difference between dyads in the MTSD-user and non-user groups in the *shared fun* and *collaboration* component. A high score on *shared fun* indicates that both the parent and the child laughed frequently during the play sessions, and the parent initiated new activities often in the Free play session. While laughter can be indicative of warmth in the relationship, it can also indicate embarrassment, due to feeling observed or showing lower skillfulness in the “Etch-a-sketch” game, which introduces uncertainty in interpreting this component. Additionally, the high loading of parental action initiation further complicates the interpretation.

A high score on *collaboration* suggests that both the parent and the child scrolled the buttons of the “Etch-a-sketch” game parallel, rather than just the child scrolling it. It is expected that *collaboration* would be higher in the non-user group, indicating higher levels of cooperation. However, the interpretation of this component is also ambiguous because alternate scrolling, where the child and the parent take turns scrolling in a synchronized manner, can also indicate cooperation. Unfortunately, our coding and analyzing methods did not allow us to assess alternate scrolling, which should be considered in future studies.

### 4.3 Problems related to MTSD use and the quantity of parent–child interactions

The importance of shared offline activities is also highlighted by our results on problems related to MTSD use. Both the scale of *conflict about MTSD use* and *problematic MTSD use* showed lower scores among MTSD-users when children shared more time with their parents offline. Children frequently interacting with their parents generally have fewer behavioral and peer-relationship problems (74), but based on a study by Beyens and Beullens (46), children co-using digital devices with parents also have less conflicts about media use compared to children who use MTSDs alone (in our case, time spent with shared online activities was unrelated to problematic MTSD use). This suggests that good parent–child relationship can be a protective factor against problematic media use, while a disadvantageous family environment can increase the chances for more frequent and serious problems related to MTSD use [for a review see (54)]. The result also highlights that MTSD use in young children may be unfavorable only if it substitutes (takes time away from) good-quality offline interactions with parents.

Interestingly, parent's education was also associated positively with problematic MTSD use (both scales). Highly educated parents might be more concerned with their child's MTSD use resulting in more conflicts and higher awareness of the child's problematic media use. In accordance with the results, higher educated parents control more their children's internet use (101), and MTSD use (29) than lower educated parents.

### 4.4 Associations between digital media use of the parent and the child

Our results supported our hypothesis that media consumption in parents and children are associated. This result is in line with previous studies showing that parents function as a role model for children in digital media use (29, 31, 32, 81). In addition, parents with a positive attitude about MTSDs also tend to encourage their children to use them (107). Finally, it is also possible that parents' heavy MTSD use takes time away from shared activities with their children, and children end up using MTSD as well. As we mentioned earlier, problematic MTSD-use could concern also adults' life with consequences on their well-being and relationships (108–110).

### 4.5 Limitations

One limitation of the study is that it only examines associations and cannot establish causality regarding the parent and child's digital media use and their interaction quantity and quality.

Another limitation is that both the free play and structured play settings in the study were designed with offline activities, and no digital activities were included. The addition of a shared online task could have provided insights into whether interaction patterns differ between online and offline tasks, as observed in previous studies (111). However, introducing an online task may raise ethical considerations, particularly in the non-user group where parents may have reservations about digital media use. Additionally, the lack of experience with digital media in the non-user group could potentially impact the evaluation of the test, as it may be perceived as highly interesting and exciting by the child.

On the other hand, the “Etch a sketch” game contains a screen, and the image displayed there changes as a result of the users' actions, which is very similar to what happens on the screen of digital devices. Therefore, MTSD-users might have advantage on this game, and this might have influenced the results (e.g., the child has to ask fewer question, the parent has to exert less control, etc.). Although the inclusion of the free play session with screen-free toys reduces the likelihood of this explanation, future studies should clarify this issue more systematically.

Although the two tasks offered in the structured play session were aimed to be of similar kind and difficulty, the results revealed a difference in the interactivity parameter during the execution of these tasks. It is possible that the two tasks differed in difficulty, but this disparity could not affect the results, as the distribution of tasks was balanced between users [24:23] and non-users [12:13].

Furthermore, a limitation of the Parent–Child Interaction Task (PCIT) and the Digital Media Use Questionnaire (DMUQ) used in the study is that they have not yet been validated. To use these measures in future studies, a validation process is necessary to establish their reliability and validity.

Further research, including longitudinal studies and validated measurement tools, is needed to provide a more comprehensive understanding of the relationship between parent–child interactions and digital media use.

## 5 Conclusion

Our study findings suggest that childhood digital media use is associated with reduced quantity and quality of interactions with parents. The decrease in quality time spent together could also increase conflicts related to MTSD use and a higher likelihood of problematic MTSD use. This study highlights the importance of considering the child's media use as a component when investigating the quality of parent–child interactions both in scientific research and also in the applied sciences like psychology or pedagogy, as the presence of MTSDs can have an impact even during the preschool years.

Furthermore, our results indicate that problematic MTSD use is a family-wide concern, as parents' MTSD use was associated with their children's MTSD use. This issue should be treated seriously, considering that the family serves as the primary social platform for a child and significantly influences the quality of future relationships. Therefore, managing early MTSD use requires a systematic approach that supports not only the focal child but also other family members.

Finally, parents' attention should be drawn to the fact that engaging in joint offline activities with their children are important not only in promoting communication and strengthening social relationships, but also in decreasing the chance of problematic MTSD use of the child. Parents' role in demonstrating responsible and mindful media use should be also highlighted. By implementing these strategies, parents can effectively navigate the challenges posed by digital media and cultivate a healthy and enriching environment for their children's development.

## Data Availability

The raw data supporting the conclusions of this article will be made available by the authors, without undue reservation.
