# Supplementary material for: Association between the use of mobile touchscreen devices and the quality of parent-child interaction in preschoolers
Source: Front Child Adolesc Psychiatry. 2024 Mar 27;3:1330243. doi: 10.3389/frcha.2024.1330243 (PMC11748892; doi:10.3389/frcha.2024.1330243)
Supplement: Supplementary file 1 [file Datasheet1.docx]

Supplementary Material

Association between the use of mobile touchscreen devices and the quality of parent-child interaction in preschoolers

**Krisztina Liszkai-Peres*, Zsófia Budai, Adrienn Kocsis, Zsolt Jurányi, Ákos Pogány, György Kampis, Ádám Miklósi, Veronika Konok**

*** Correspondence:**

**Krisztina Liszkai-Peres**

peres.krisztina@ppk.elte.hu

# Screening questionnaire (before participation in the study)

Parent’s data

1. Your name:
2. Your email address:

Child’s data

1. First name of the child:
2. Child’s date of birth:
3. Gender of the child:
   - Boy
   - Girl
   - Other
4. Which of the following statements is characteristic of your child?

[In this section, we are asking whether your child uses touchscreen mobile devices, such as smartphones and tablets, and if so, how frequently. We distinguish between active usage (e.g., when your child starts an application or video, or when they play games on the device or take photos) and passive usage (e.g., when your child merely watches a video initiated by you).]

- - They have never used a smartphone or tablet (not even for watching cartoons) or used smart devices less than five times in their lives.
  - They occasionally watch cartoons/videos on a smartphone or tablet, but they have never used such devices actively (e.g., they don't play games, start/operate videos, take photos, etc.).
  - Sometimes they use smartphone or tablet actively, but not regularly (up to once a month at most).
  - They use smartphone or tablet (actively) a couple of times in a month.
  - They use smartphone or tablet (actively) on a weekly basis.
  - They use smartphone or tablet (actively) every day or almost every day.

1. How long has your child been using a touchscreen device (smartphone or tablet)?
   - For less than 6 months.
   - For more than 6 months.
2. Roughly how much time does your child spend with touchscreen devices (smartphone and tablet) on a WEEKLY basis? [If your child uses both a smartphone and a tablet, provide the combined duration of both activities.]
3. Does your child play digital games on a smartphone/tablet?
   - Yes
   - No
4. Does your child have their own smartphone/tablet (or a device they share with siblings)?
   - Yes
   - No
5. What operating system is running on your child's smartphone/tablet?
   - Android
   - iOS (iPhone or iPad)
6. If your child uses your (or another parent's) smartphone or tablet, what operating system is running on it?
   - Android
   - iOS (iPhone or iPad)

Certain factors can affect participation in the research. For example, if your child has any psychiatric disorder. Please answer the following questions.

1. Has your child been diagnosed with any developmental or psychiatric disorder?
   - Yes
   - No

[If yes:]

1. If you know the exact name(s) and/or code(s) of the diagnosis(es), please write them here:
2. If you can assign the diagnosed problem(s) into the following categories, please select the appropriate one(s). (If your child received multiple diagnoses that belong to different categories, you can select more than one.)
   - Anxiety disorders (e.g., generalized anxiety disorder, obsessive-compulsive disorder, separation anxiety, phobias, panic, etc.).
   - Behavioral disorders (e.g., conduct disorder, oppositional defiant disorder).
   - Attention and activity disorders (e.g., ADHD - Attention-Deficit/Hyperactivity Disorder, with or without hyperactivity).
   - Pervasive developmental disorders (e.g., autism, Asperger's syndrome, autism spectrum disorder, Rett syndrome).
   - Eating disorders (e.g., rumination, pica, anorexia, bulimia).
   - Elimination disorders (e.g., enuresis - bedwetting, encopresis - involuntary bowel movements).
   - Childhood emotional disorders (e.g., childhood depression).
   - Tic disorders (e.g., tics, Tourette syndrome).
   - Speech disorders (e.g., aphasia, dysphasia, stuttering).
   - Socialization disorders (e.g., elective mutism, reactive attachment disorder).
   - Intellectual disability.
   - I cannot categorize it.
   - Other:

# Digital Media Use Questionnaire (DMUQ)

## Data of the family

1. Your age:
2. Your gender:

- male
- female
- other
- I do not want to answer this question

1. Highest level of education:

- Elementary/primary school
- Associate's degree: occupational, technical or vocational program
- High School Graduate
- Bachelor’s degree (BA/BSc)
- Master’s degree (MA/MSc)
- Doctoral degree (PhD)

1. Number of adults living in your household:
2. Number of children living in your household:
3. Regarding the child participating in the study, where does the child stand in the sibling order?
4. Does your child attend preschool?
5. Monthly net income of the family:
6. How many devices do you have at home from the list below:

- TV
- laptop / PC
- smartphone
- smartwatch
- tablet
- console (e.g. xbox, playstation)

1. How much time do you spend watching TV in the presence of your child daily?
2. How much time do you spend using a smartphone in the presence of your child daily?
3. How much time do you spend using a laptop/PC in the presence of your child daily?
4. How much time do you spend using a tablet in the presence of your child daily?

## Digital device use of your child

1. Please mark the devices that your child usually uses and can to use independently.

- TV
- laptop / PC
- smartphone
- smartwatch
- tablet
- console (e.g. Xbox, PlayStation)
- none of them

1. How much time does your child spend watching TV daily?
2. How much time does your child spend using a laptop or PC daily?
3. Does your child use a smartphone or a tablet?

- Yes
- No (continue with Question 26)

1. How much time does your child spend using a smartphone daily?
2. How much time does your child spend using a tablet daily?
3. How much time does your child spend watching videos/cartoons on a smartphone/tablet daily?
4. How much time does your child spend playing games on a smartphone/tablet daily?
5. Please mark the activities your child usually does on the smartphone or tablet.

- watching videos/cartoons
- playing games
- watching photos
- listening to music
- taking photos
- phone calls / video chatting
- other

## Problematic mobile/tablet use

1. How often do the following events occur in your family? (1 – never, 2 – less than once a month, 3 – 1-3 times a month, 4 – 1-3 times a week, 5 – 4-6 times a week, 6 – every day)

- You have to ask your child to stop mobile/tablet use. (Because he/she has used it for a long time or has something else to do.)
- Mobile/tablet use generates conflicts between the child and you/other parent.
- There is a conflict/quarrel/tantrum because the child has to stop using mobile/tablets.
- There is a conflict/quarrel/tantrum because the child wants to use mobile/tablets but you/other parent do not allow it.

1. How much do you agree with the statements below? (1 –strongly disagree, 2 – somewhat disagree, 3 – neither agree or disagree, 4 – somewhat agree, 5 – strongly agree)

- My child has difficulties to stop mobile/tablet use.
- My child becomes tense/nervous/angry when they have to stop using mobile/tablet.
- My child wants to use mobile/tablet all the time.
- My child shows signs of mobile/tablet use addiction.

## Shared free time activities

1. Which digital activities do you do together with your child?

- playing digital games
- watching a tv/movie/cartoon
- reading an e-book/digital content
- taking a photo/drawing/other creative activity on a smartphone/tablet
- making phone calls / video chat with friends, relatives
- other

1. How much time do you spend with digital activities together with your child on a typical weekday?
2. How much time do you spend with digital activities together with your child on a typical weekend day?
3. Which nondigital activities do you do together with your child?

- playing with toys
- reading stories
- creative activity (e.g. drawing, playing with playdough, playing with building blocks)
- outdoor activities (e.g. ride a bicycle, go for a walk, hiking)
- cultural activities (e.g. theatre, concert)
- shopping (e.g. grocery, shopping mall)
- go to the restaurant, confectionery, ice cream shop
- meeting with friends, relatives

1. How much time do you spend with nondigital activities together with your child on a typical weekday?
2. How much time do you spend with nondigital activities together with your child on a typical weekend day?

# Coded variables of parent-child interaction

Variables coded during the Parent-Child Interaction tasks:

|  | Variable name | Definition | Instant/continuous |
| --- | --- | --- | --- |
| Free play | Physical contact | Physical contact between parent's and child's body | Continuous |
|  | Attention towards parent | Child looks at parent | Continuous |
|  | Attention towards child | Parent looks at child | Continuous |
|  | Attention by parent | Parent calls child's attention to something (verbal/nonverbal) | Instant |
|  | Attention by child | Child calls parent's attention to something (verbal/nonverbal) | Instant |
|  | Joint attention | Child and parent are looking at the same object | Continuous |
|  | Shared play | Child and parent are playing together | Continuous |
|  | Praise | Praise/positive verbal utterance by the parent | Instant |
|  | Scolding | Scolding/criticism/negative verbal remark by the parent | Instant |
|  | Parent laughs | Parent laughs | Continuous |
|  | Child laughs | Child laughs | Continuous |
|  | Child asks | Child asks a verbal question or for help | Instant |
|  | Help | Parental response to child’s request for help (verbal and nonverbal) | Instant |
|  | No help | No parental response to child’s request for help | Instant |
|  | Answer | Parental response to child’s question | Instant |
|  | No answer | No parental response to child’s question | Instant |
|  | Action by parent | The parent initiates a new activity (verbal/nonverbal) | Instant |
|  | Action by child | The child initiates a new activity (verbal/nonverbal) | Instant |
|  | Physical control | Physical control by parent e.g. parent grabs the child's hand to stop an action | Instant |
| Structured play | Praise | Praise/positive verbal remark by the parent | Instant |
|  | Scolding | Scolding/criticism/negative remark by the parent | Instant |
|  | Parent laughs | Parent laughs | Continuous |
|  | Child laughs | Child laughs | Continuous |
|  | Child asks | Child asks verbal question or for help | Instant |
|  | Help | Parental response to child’s request for help (verbal/nonverbal) | Instant |
|  | No help | No parental response to child’s request for help | Instant |
|  | Answer | Parental response to child’s question | Instant |
|  | No answer | No parental response to child’s question | Instant |
|  | Parent instructs | Instruction/guidance by parent | Continuous |
|  | Child instructs | Instruction/guidance by child | Continuous |
|  | Parent scrolls | Parent alone handles etch-a-sketch | Continuous |
|  | Child scrolls | Child alone handles etch-a-sketch | Continuous |
|  | Both scrolls | Both handle etch-a-sketch | Continuous |
|  | Physical control | Physical control by parent e.g. parent grabs the child's hand to stop an action | Instant |

# Demographic characteristics of MTSD-users and non-users

| **MTSD-users** | | | | |
| --- | --- | --- | --- | --- |
|  | | | Statistic | Std. Error |
| Child age | Mean | | 5.362 | .123 |
|  | 95% Confidence Interval for Mean | Lower Bound | 5.113 |  |
|  |  | Upper Bound | 5.611 |  |
|  | 5% Trimmed Mean | | 5.354 |  |
|  | Median | | 5.2648 |  |
|  | Variance | | .623 |  |
|  | Std. Deviation | | .789 |  |
|  | Minimum | | 4.087 |  |
|  | Maximum | | 6.806 |  |
|  | Range | | 2.718 |  |
|  | Interquartile Range | | 1.341 |  |
|  | Skewness | | .122 | .369 |
|  | Kurtosis | | -1.032 | .724 |
| Parent age | Mean | | 38.76 | .861 |
|  | 95% Confidence Interval for Mean | Lower Bound | 37.02 |  |
|  |  | Upper Bound | 40.50 |  |
|  | 5% Trimmed Mean | | 38.78 |  |
|  | Median | | 39.00 |  |
|  | Variance | | 30.389 |  |
|  | Std. Deviation | | 5.513 |  |
|  | Minimum | | 28 |  |
|  | Maximum | | 49 |  |
|  | Range | | 21 |  |
|  | Interquartile Range | | 10 |  |
|  | Skewness | | .019 | .369 |
|  | Kurtosis | | -.802 | .724 |
| Parent education | Mean | | 3.07 | .176 |
|  | 95% Confidence Interval for Mean | Lower Bound | 2.72 |  |
|  |  | Upper Bound | 3.43 |  |
|  | 5% Trimmed Mean | | 3.08 |  |
|  | Median | | 3.00 |  |
|  | Variance | | 1.270 |  |
|  | Std. Deviation | | 1.127 |  |
|  | Minimum | | 1 |  |
|  | Maximum | | 5 |  |
|  | Range | | 4 |  |
|  | Interquartile Range | | 2 |  |
|  | Skewness | | .071 | .369 |
|  | Kurtosis | | -.639 | .724 |
| Family net income | Mean | | 528829.27 | 33075.743 |
|  | 95% Confidence Interval for Mean | Lower Bound | 461980.70 |  |
|  |  | Upper Bound | 595677.84 |  |
|  | 5% Trimmed Mean | | 520379.40 |  |
|  | Median | | 500000.00 |  |
|  | Variance | | 4.485E + 10 |  |
|  | Std. Deviation | | 211788.090 |  |
|  | Minimum | | 180000 |  |
|  | Maximum | | 1100000 |  |
|  | Range | | 920000 |  |
|  | Interquartile Range | | 260000 |  |
|  | Skewness | | .861 | .369 |
|  | Kurtosis | | .140 | .724 |

| **Non-users** | | | | |
| --- | --- | --- | --- | --- |
|  | | | Statistic | Std. Error |
| Child age | Mean | | 5.179 | .144 |
|  | 95% Confidence Interval for Mean | Lower Bound | 4.880 |  |
|  |  | Upper Bound | 5.479 |  |
|  | 5% Trimmed Mean | | 5.144 |  |
|  | Median | | 5.028 |  |
|  | Variance | | .457 |  |
|  | Std. Deviation | | .675 |  |
|  | Minimum | | 4.208 |  |
|  | Maximum | | 6.847 |  |
|  | Range | | 2.639 |  |
|  | Interquartile Range | | 1.071 |  |
|  | Skewness | | .765 | .491 |
|  | Kurtosis | | .181 | .953 |
| Parent age | Mean | | 36.73 | .881 |
|  | 95% Confidence Interval for Mean | Lower Bound | 34.90 |  |
|  |  | Upper Bound | 38.56 |  |
|  | 5% Trimmed Mean | | 36.74 |  |
|  | Median | | 37.50 |  |
|  | Variance | | 17.065 |  |
|  | Std. Deviation | | 4.131 |  |
|  | Minimum | | 30 |  |
|  | Maximum | | 43 |  |
|  | Range | | 13 |  |
|  | Interquartile Range | | 8 |  |
|  | Skewness | | .076 | .491 |
|  | Kurtosis | | -1.337 | .953 |
| Parent education | Mean | | 3.77 | .207 |
|  | 95% Confidence Interval for Mean | Lower Bound | 3.34 |  |
|  |  | Upper Bound | 4.20 |  |
|  | 5% Trimmed Mean | | 3.85 |  |
|  | Median | | 4.00 |  |
|  | Variance | | .946 |  |
|  | Std. Deviation | | .973 |  |
|  | Minimum | | 1 |  |
|  | Maximum | | 5 |  |
|  | Range | | 4 |  |
|  | Interquartile Range | | 1 |  |
|  | Skewness | | -.865 | .491 |
|  | Kurtosis | | 1.690 | .953 |
| Family net income | Mean | | 617272.73 | 54324.589 |
|  | 95% Confidence Interval for Mean | Lower Bound | 504298.56 |  |
|  |  | Upper Bound | 730246.89 |  |
|  | 5% Trimmed Mean | | 603535.35 |  |
|  | Median | | 550000.00 |  |
|  | Variance | | 6.493E+10 |  |
|  | Std. Deviation | | 254804.908 |  |
|  | Minimum | | 300000 |  |
|  | Maximum | | 1200000 |  |
|  | Range | | 900000 |  |
|  | Interquartile Range | | 425000 |  |
|  | Skewness | | .658 | .491 |
|  | Kurtosis | | -.467 | .953 |

# Normality tests of demographic and media use data of MTSD-users and non-users

i. MTSD-users

The results of the Shaphiro-Wilk tests showed that both children’ and parents’ age were normally distributed (age of children W_46_ = .965, p = .183; parents’ age W_46_ = .974, p = .399). However, neither parent education (W_46_ = .911, p = .002), nor family net income (W_42_ = .929, p = .012) was normally distributed.

Regarding the media use of children none of the investigated variables were normally distributed, neither separately (daily TV: W_48_ = .916, p = .002; daily mobile: W_48_ = .690, p < .001; daily tablet: W_48_ = .811, p < .001), nor as a summarized variable (total MTSD: W_48_ = .840, p < .001; total media consumption: W_48_ = .925, p = .005).

Similarly, the investigated variables of parents’ media use were not normally distributed, neither separately (daily TV: W_47_ = .812, p < .001; daily mobile: W_47_ = .840, p < .001; daily tablet: W_47_ = .396, p < .001; daily laptop W_47_ = 0.705, p < .001), nor as a summarized variable (total MTSD: W_47_ = .782, p < .001; total media consumption: W_47_ = .919, p = .003).

ii. Non-users

Shaphiro-Wilk tests showed that children’ and parents’ age were normally distributed (age of children W_24_ = .937, p = .142; parents’ age W_24_ = .935, p = .128), and also monthly net income showed normal distribution (W_22_ = .930, p = .122). However, parent education (W_24_ = .847, p = .002) was not normally distributed.

Regarding screen time we could only analyze daily TV watching, because the inclusion criteria for non-users was not to use MTSDs. This variable was not normally distributed (W_22_ = .775, p < .001).

Regarding parents’ media use none of the investigated variables were normally distributed, neither separately (daily TV: W_25_ = .651, p < .001; daily mobile: W_25_ = .737, p < .001; daily tablet: W_25_ = .439, p < .001; daily laptop W_25_ = .772, p < .001), nor as a summarized variable (total MTSD: W_25_ = .761, p < .001; total media consumption: W_25_ = .856, p = .002).

# PCA of parent-child interaction

Item loadings on the principal components of parent-child interaction and the percentage of variance explained by the components. Free play (‘fp’) and structured play (‘sp’) are indicated in brackets.

|  | Component | | | | |
| --- | --- | --- | --- | --- | --- |
|  | Shared fun (17.82%) | Interactivity (13.47%) | Parental control (11.69%) | Attention towards partner (10.39%) | Collaboration (8.55%) |
| Looking towards parent (fp) |  | 0.158 | -0.116 | 0.642 | -0.287 |
| Action by parent (fp) | 0.473 | 0.221 | 0.129 |  |  |
| Attention by parent (fp) |  |  | 0.638 |  | -0.115 |
| Attention by child (fp) | -0.132 | 0.498 | -0.110 | 0.256 | 0.224 |
| Answer (fp) |  | 0.790 |  | 0.251 |  |
| Physical control (fp) | -0.119 |  | 0.899 |  | 0.140 |
| Praise (sp) | 0.188 | 0.560 | 0.390 |  | -0.279 |
| Child asks (sp) |  | 0.800 |  |  |  |
| Physical control (sp) | -0.109 |  | 0.848 |  | 0.179 |
| Looking towards child (fp) |  |  | -0.144 | 0.765 | 0.141 |
| Joint attention (fp) | 0.260 | 0.173 |  | -0.720 | -0.128 |
| Parent laughs (fp) | 0.894 |  |  | 0.137 |  |
| Child laughs (fp) | 0.835 |  | -0.108 |  |  |
| Parent laughs (sp) | 0.824 |  |  |  | 0.319 |
| Child laughs (sp) | 0.793 | -0.128 |  | -0.208 | 0.180 |
| Parent instructs (sp) | 0.272 | 0.168 | 0.298 | 0.640 | -0.118 |
| Child scrolls (sp) |  |  |  |  | -0.836 |
| Parent scrolls (sp) | 0.310 | 0.474 | 0.133 | -0.288 | -0.131 |
| Both scroll (sp) | 0.184 | 0.139 | 0.120 |  | 0.793 |
| Child instructs (sp) |  | 0.550 | -0.115 | -0.242 |  |
